# The Microbiota Architecture of the Chinchilla Gastrointestinal Tract

**DOI:** 10.3390/vetsci11020058

**Published:** 2024-01-28

**Authors:** Yuhong Wu, Bo Liu, Xinyi Ma, Luo Yang, Xinyi Lu, Wei Wang, Jing Li

**Affiliations:** 1 Department of Clinical Veterinary Medicine, College of Veterinary Medicine, China Agricultural University, Beijing 100193, Chinabliu2017@cau.edu.cn (B.L.); lironyoung@cau.edu.cn (L.Y.); morri_lu05@cau.edu.cn (X.L.);; 2 Veterinary Teaching Hospital, China Agricultural University, Beijing 100193, China

**Keywords:** chinchillas, gastrointestinal microbiota, 16S rRNA

## Abstract

**Simple Summary:**

Chinchillas (*Chinchilla lanigera*) are cecal fermenters; the health of their gastrointestinal system contributes significantly to their overall wellbeing, yet minimal data are available regarding the composition of the microbiota present in different segments of their gastrointestinal tract. This study addresses the knowledge gap regarding the gastrointestinal microbiota in healthy chinchillas. Notably, the cecum and colon displayed significantly higher microbiota abundance compared with the proximal gastrointestinal segments, with cellulose-degrading bacteria as the core microbiota. In contrast, the stomach and small intestine exhibited lower microbiota abundance, with characteristic microbiota possessing acid-resistant properties. Moreover, the Atopobiaceae family was discovered in the proximal gastrointestinal tract of the chinchilla, which is the first report of its presence in the gastrointestinal tract of cecal fermenters.

**Abstract:**

The gastrointestinal microbiota develop alongside the host and play a vital role in the health of cecal fermenters such as chinchillas. However, little is known about the microbiota architecture in healthy chinchillas. Illumine-based 16S rRNA gene amplicon sequencing was used to investigate the microbiota present in six different gastrointestinal tract regions of three healthy adult chinchillas. The findings revealed significantly more abundant microbiota in the large intestine compared with the proximal segments. In addition, the cecum exhibited better evenness compared to the colon. The core microbiota are Firmicutes, Bacteroidota, Actinobacteriota, and Proteobacteria at the phylum level. The signature microbiota of each segment were identified. The cecum had 10 signature microbiota, which had the widest coverage and overlapped with that of the cecum. The stomach had five signature microbiota, exhibiting the second widest coverage and overlapping with the duodenum. No signature microbiota were detected in the jejunum and ileum. While similarities exist with the microbiota of other cecal fermenters, chinchillas exhibit distinct microbiota closely related to their unique digestive mechanisms. This study is a preliminary study of the gastrointestinal microbiota architecture and distribution in healthy chinchillas. Further study is needed in order to better understand the effect of gastrointestinal microbiota on the health of the chinchilla.

## 1. Introduction

Chinchillas are cecal fermenters with a unique gastrointestinal digestive system. Chinchillas have a large and well-developed cecum that accounts for up to 22% of the total gastrointestinal tract, and chinchillas rely on the microbiota within the cecum to ferment plant fibers and produce volatile fatty acids (VFAs) as a crucial energy source. Chinchillas practice coprophagy. The mucus on the surface of cecotropes protects the microorganisms within it from the gastric acid, which allows these microorganisms to return to the gastrointestinal tract with minimal loss [1,2,3,4].

“Gastrointestinal microbiota” refers to the entire bacterial community residing in an animal’s gastrointestinal tract, which maintains a symbiotic relationship with the mucosal lining. The microbiota are believed to be able to provide insights into the health status of the animals [1]. In healthy chinchilla, the gastrointestinal microbiota play crucial roles in the host’s metabolisms, immune responses, and anti-inflammatory functions, serving as a vital defense barrier against invasive pathogenic microorganisms [1,5,6]. Minimal data are available regarding the composition of the microbiota present in the chinchilla gastrointestinal tract. O’Donnell et al. have studied the core fecal microbiota of three chinchillas living on a farm in Ireland along with nine other animal species. The result of the study showed that the Firmicutes and Bacteroidetes phyla were the dominant microbiota in the chinchilla [6]. Our team evaluated the effect of ivermectin treatment on the fecal bacterial microbiota of healthy chinchillas [7]. In addition to the effect of gastrointestinal drugs on the fecal microbiota, differences in microbial composition between compartments of the chinchilla gastrointestinal tract are also of clinical interest. The present study investigates microbial composition and distribution in different segments of the chinchilla gastrointestinal tract via 16S rRNA gene amplicon sequencing and is therefore a valuable reference for further research on chinchilla microbiota and potential clinical applications thereof in diagnosis and treatment.

## 2. Materials and Methods

### 2.1. Animal Selection

Three healthy adult chinchillas from a commercial farm in Beijing were selected in this study, including one male and two female chinchillas, aged between one to two years old. All the chinchillas had not received any drugs, especially antibiotics, for six months before this experiment. The chinchillas were housed in individual cages at consistent temperatures of 21–23 °C, and the light was present for 12 h a day. All the animals were fed a commercial pellet feed (Jolly^®^ Chinchilla Fullvit JP05, Double Trees Company Ltd., Dongguan, China) and tap water ad libitum. The commercial pallet feed was composed of rosehip, corn, soybean, evening primrose powder, yeast powder, salt, and various vitamins. Additionally, they were provided with apple tree branch for teeth grinding every two days and given sand baths every week. All animals had been healthy during the experiment based on the results of physical examination, appetite, urination, and feces consistency. This experiment was approved by the Experimental Animal Welfare and Animal Experiment Ethics Review Committee of China Agricultural University. 

### 2.2. Sample Collection

All animals were anesthetized with 0.5 mg/kg butorphanol and 5 mg/kg alfaxalone intramuscular injection. Euthanasia of these three chinchillas via cervical dislocation was performed after they became unconscious. The abdomen was clipped and prepped in the standard sterile fashion. The stomach, duodenum, jejunum, ileum, cecum, and colon were excised using sterile dissecting instruments. Approximately 1–2 mL of gastrointestinal contents were collected through gently scooping the gastrointestinal content with a sterile scalpel [8]. All samples were collected within 30 min after euthanasia. All samples were placed in sterile 2 mL cryogenic tubes and stored at −80° C before being sent for inspection.

### 2.3. DNA Extraction and 16S rRNA Sequencing 

Bacterial genomic DNA was extracted from gastrointestinal content samples using a commercial extraction kit (E.Z.N.A.^®^ DNA Extraction Kit, Norcross, GA, USA: Omega Bio-Tek), according to the manufacturer’s recommendations. The quality of the extracted DNA was detected by 1% agarose gel electrophoresis, and its concentrations were determined by a NanoDrop2000 ultramicro spectrophotometer. The V3 and V4 hypervariable region of the 16S rRNA gene was amplified using the upper primer 338F (5′-ACTCCTACGGGAGGCAGCAG-3′) and the lower primer 806R (5′-GGACTACHVGGGTWTCTAAT-3′). The amplicons were then sequenced using Illumina Miseq PE300.

### 2.4. Statistical Analysis

The 16S rRNA raw amplicon sequences obtained from each sample were merged using FLASH (v1.2.11). All bioinformatic analyses were conducted using the USEARCH pipeline (v11), clustering filtered reads into Operational Taxonomic Units (OTUs) at 97% nucleotide identity level. Normalization was performed to the OUT matrices to a total of 20,000 counts. Taxonomic assignments were performed via the RDP classifier using 0.5 as the confidence threshold, against the Silva 16S rRNA database (v138). The QIIME pipeline was used to generate a table of the abundance in each taxonomic group. 

For α-diversity analysis, the richness of OTUs was assessed using the Chao 1 and the ACE estimator. In addition, the Simpson index and the Shannon index were used for assessing the evenness. β-diversity analysis was performed via Bray–Curtis similarity indices and visualized using principal coordinates analysis (PCoA) and nonmetric multidimensional scaling (NMDS). Finally, linear discriminant analysis effect size (LEfSe) was used to determine the marker flora of each sample according to the all-against-all multigroup comparison strategy with LDA > 4 and *p* < 0.05 as the standard. The difference among different gastrointestinal segments was tested by the Kruskal–Wallis rank sum test. The Welch *t*-test was applied to all samples to determine whether the differences in the microbiota among the segments were statistically significant. A *p*-value of less than 0.05 was considered statistically significant. 

## 3. Results

### 3.1. Microbiota Profile Analysis

**Figure 1:**
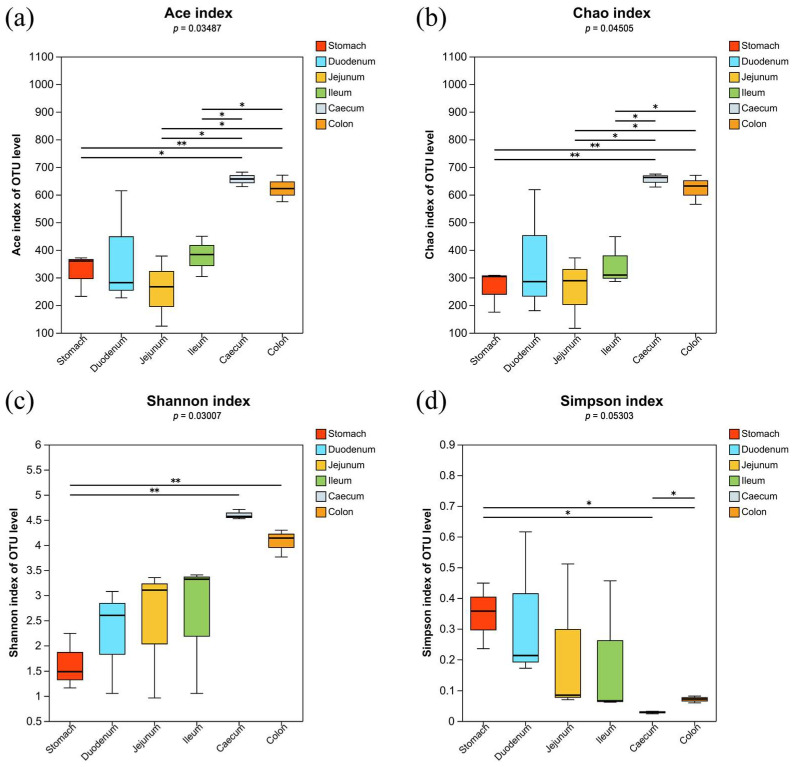
Gastrointestinal microbiota α-diversity analysis for different gastrointestinal segments: (**a**) ACE index, (**b**) Chao index, (**c**) Shannon index, and (**d**) Simpson index. One asterisks (*) means *p* < 0.05. Two asterisks (**) means *p* < 0.01.

All samples of chinchilla gastrointestinal content contained a total of 1120 bacterial OTUs; among them, the out numbers of the stomach, duodenum, jejunum, ileum, cecum, and colon group were 304, 665, 413, 492, 841, and 802, respectively. The microbiota in the large intestine, including in the cecum and the colon, exhibited the highest richness and showed significant difference to the proximal segments (*p* < 0.05). The large intestine showed the most diverse microbiota, and there was significant difference between the large intestine and the stomach (*p* < 0.05). In addition, there was significant difference in the diversity between the cecum and the colon (*p* < 0.05, Figure 1).

**Figure 2:**
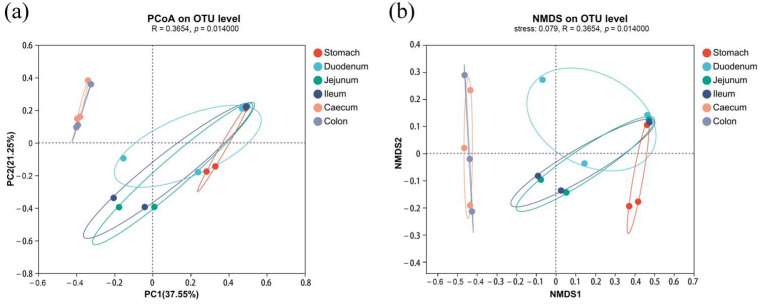
Gastrointestinal microbiota β-diversity analysis between different segments: (**a**) PCoA analysis and (**b**) NMDS analysis.

The microbiota of the 18 samples were classified into two categories: the large intestine (cecum and colon) and the proximal gastrointestinal tract (stomach, duodenum, jejunum, and ileum). The PCoA results revealed that there was greater difference between different segments than within the same segments (R = 0.37, *p* = 0.014). In addition, the microbiota in the large intestine were significantly different to the proximal gastrointestinal tract (*p* < 0.05). In this experiment, the stress value of NMDS was 0.079, indicating a meaningful interpretation of the NMDS analysis and a significant difference between the large intestine and the proximal gastrointestinal tract (*p* < 0.05) (Figure 2). Both PCoA and NMDS analyses consistently demonstrated a high similarity in the microbiota of the cecum and colon. The elliptical ranges of stomach, duodenum, jejunum, and ileum overlapped, suggesting no significant difference among these different segments. The microbiota of the jejunum and ileum showed high similarity but were distinct from those of the stomach and duodenum.

### 3.2. Compositional Analysis 

**Figure 3:**
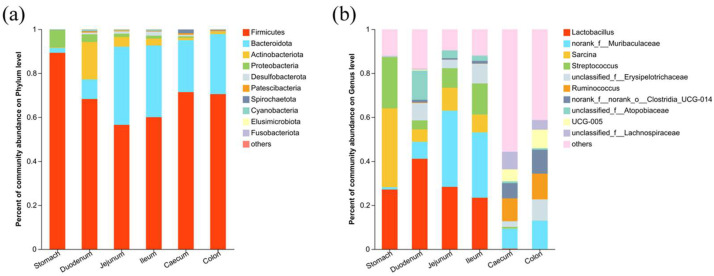
Taxa composition of different segments of the GI tract in chinchillas: (**a**) microbial composition at phyla level and (**b**) microbial composition at genera level.

The abundance of taxa in all different gastrointestinal content samples were analyzed at both the phylum level and genus level. Four major phyla (Firmicutes, Bacteroidota, Actinobacteriota, and Proteobacteria) collectively accounted for more than 95% of the relative abundance. Firmicutes were the most abundant phylum across all gastrointestinal segments, with the stomach exhibiting the highest abundance (89.34%) among all segments. In the duodenum, Actinobacteriota were the second dominant phylum. In the jejunum, ileum, cecum, and colon, Bacteroidota had the second highest relative abundance (Figure 3a). 

The top 10 abundant microbiota bacterial genera in the gastrointestinal tract of chinchillas were Lactobacillus, Muribaculaceae, Sarcina, and Streptococcus, Erysipelotrichaceae, Ruminococcus, Clostridia_UCG-014, Atopobiaceae, and Lachnospiraceae. Among these genera, Lactobacillus, Sarcina, and Streptococcus accounted for 86.29% of the bacteria in the stomach. This decreased to greater than 1% in the small intestine and less than 1% in the large intestine, respectively. In the duodenum, Lactobacillus was the most dominant genus, followed by Atopobiaceae, together comprising 54.38% of the total duodenal microbiota. Both the jejunum and ileum were dominated by Muribaculaceae and Lactobacillus (Figure 3b). In the large intestine, the Muribaculaceae and Ruminococcus were the dominate genera. The abundance of Muribaculaceae (*p* < 0.05) in the large intestine was significantly higher than that in the stomach. The abundance of Ruminococcus in the large intestine was significantly higher than in the proximal segments (*p* < 0.05). 

### 3.3. Gastrointestinal Signature Microbiota

In this experiment, the cecum signature microbiota had the widest coverage, followed by the stomach. The colon signature microbiota overlapped with that of the cecum, and the duodenum signature microbiota overlapped with that of the stomach. However, no signature microbiota were detected in the jejunum and ileum.

**Figure 4:**
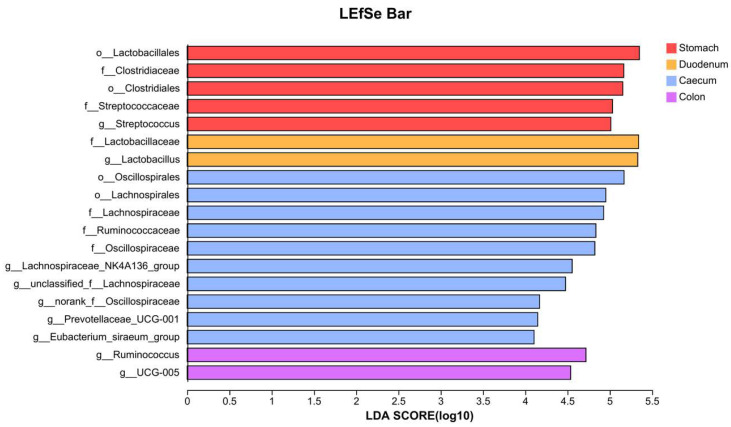
LDA–LEfSe analysis for microbiota in different segments of the gastrointestinal tract.

Nineteen taxa were identified to be associated with different gastrointestinal locations (LDA > 4, *p* < 0.05), including four orders, six families, and nine genera (Figure 4). The cecum had 10 signature microbiota, with the order Oscillospirales contributing the most to the differences (*p* = 0.021), followed by Lachnospirales (*p* = 0.026). Besides *Prevotellaceae_UCG-001*, all the other signature families or genera belonged to either Oscillospirales or Lachnospirales. *Prevotellaceae_UCG-001* (*p* = 0.045) belonged to the order Bacteroidales and was also a signature microbiota in the cecum. 

The characteristic taxa in the colon included the genus *Ruminococcus* (*p* = 0.024) and *UCG-005* (*p* = 0.045). The stomach had a total of five signature microbiota, including the order Clostridiales (*p* = 0.03) and its family Clostridiaceae (*p* = 0.03), as well as the order Lactobacillales (*p* = 0.034) and its family Streptococcaceae (*p* = 0.039) and genus *Streptococcus* (*p* = 0.039). The characteristic taxa in the duodenum included the family Lactobacillaceae (*p* = 0.033) and the genus *Lactobacillus* (*p* = 0.033).

## 4. Discussion

This study is a pilot study to analyze the composition and distribution characteristics of the gastrointestinal microbiota in healthy chinchillas. Two clusters of bacterial populations were observed in the digestive system: (i) stomach and small intestine and (ii) large intestine. Among all the gastrointestinal segments, the cecal microbiota had the highest diversity, while the stomach exhibited the lowest diversity. The diversity of microbiota within the cecum and colon was significantly higher than in the proximal segments. Despite the similar microbiota abundances in the cecum and the colon, the cecum microbiota were more evenly distributed. Due to the mechanisms of colonic separation, the contents of the cecum and the colon may mix during intestinal peristalsis [2], which corresponds with the high diversity and similar composition of the microbiota in the cecum and colon as observed in the study.

At the phylum level, the core phyla in the gastrointestinal tract of chinchillas include Firmicutes, Bacteroidota, Actinobacteriota, and Proteobacteria. The results of this study indicate some similarity in the microbiota composition between chinchillas and other cecal fermenters like rabbits [6,9]. At the genus level, *Ruminococcus* and *Muribaculaceae* showed similar composition between chinchillas and other cecal fermenters [6,9]. However, to our knowledge, there has been no previous research on the detection of the Atopobiaceae family in the gastrointestinal microbiota of cecal fermenters. In our study, *Atopobiaceae* was found to be the second most abundant genus in the duodenum of healthy chinchillas. Atopobiaceae, which belongs to Actinobacteriota phylum, is beneficial bacteria found in the gastrointestinal tracts of other mammals such as humans, mice and pigs. Many bacteria within the Atopobiaceae family are capable of fermenting and producing lactic acid and short-chain fatty acids [10]. Some Atopobiaceae bacteria have been reported to exhibit a high bile resistance ability, which makes them potential targets for future probiotic research [10].

In our study, the characteristic microbiota in the cecum comprised three major categories: the Oscillospirales order, Lachnospirales order, and *Prevotellaceae_UCG-001* genus. This finding correlated with other studies on the microbiota of cecal fermenters [6,9]. The Ruminococcaceae family and Oscillospiraceae family belong to the Oscillospirales order. Ruminococcaceae can degrade polysaccharides to produce short-chain fatty acids as butyric acid, providing an energy source for intestinal epithelial cells and maintaining the stability of the intestinal mucosa [11]. Oscillospiraceae is involved in protein degradation and metabolism and are beneficial bacteria that produce butyric acid [12,13]. This bacterial family has the potential to degrade benzoic acid, a capability that is significantly positively correlated with resistance to parasitic infections [14]. The Lachnospiraceae family and the *Lachnospiraceae_NK4A136_group* genus within it belong to the order Lachnospirales. The Lachnospiraceae family can hydrolyze starch and other sugars and produce short-chain fatty acids as a primary source of nutrition for intestinal epithelial cells. It also plays a role in alleviating inflammation and enhancing host immunity [15]. The genus *Lachnospiraceae_NK4A136_group* consists of fiber-degrading bacteria that primarily produce butyrate. It plays a crucial role in maintaining intestinal barrier integrity, suppressing inflammation, and exhibiting anti-tumor effects [12,16]. In studies of gut microbiota in mice, the *Lachnospiraceae_NK4A136_group* has shown a significant negative correlation with intestinal permeability and plasma lipopolysaccharide levels, suggesting its positive impact on controlling obesity [17]. The *Prevotellaceae_UCG-001* is capable of digesting dietary proteins and amino acids but cannot directly participate in cellulose degradation [18,19]. In research conducted by Ma et al., *Prevotellaceae_UCG-001* was found to have a relatively low abundance in the gut of animals with colitis. It was suggested that this genus can activate the adenosine monophosphate-activated protein kinase (AMPK) signaling pathway to improve the overall intestinal health of the host [20].

In this study, the genus *Ruminococcus* and *UCG-005* were characteristic microbiota found in the colon of chinchilla, and their distribution pattern is similar to that observed in rabbits [9]. The *UCG-005* can degrade cellulose and produce metabolic products such as acetic acid and butyric acid, which are strongly positively correlated with the concentration of short-chain fatty acids in the host’s body [21,22]. Research by Chen et al. found that its abundance in the cecum of rabbits was positively correlated with the levels of IL-10 and total antioxidant capacity in the ileum [23]. Therefore, *UCG-005* may have anti-inflammatory properties [23].

The characteristic microbiota in the stomach included Clostridiales and Lactobacillales. Their dominant presence in the stomach is believed to be related to its acid tolerance [24,25,26]. The *Streptococcus* genus, belonging to the order Lactobacillales, is a common Gram-positive opportunistic pathogen [27,28]. Despite its significant associations with various gastrointestinal diseases, several studies have identified *Streptococcus* as one of the core microbial groups in the stomach and small intestine of healthy people [24,29]. In this study, the *Streptococcus* genus is one of the dominant microbial genera in the chinchilla gastrointestinal tract, particularly in the stomach and small intestine, with a distribution pattern similar to that in horses [30]. The Lactobacillaceae family, and the *Lactobacillus* genus within it, represents the characteristic microbiota found in the duodenum. Their presence was a result of their robust acid tolerance [24,31,32].

## 5. Conclusions

In conclusion, despite the limitation of a low number of animals used in the study, the present study led to a better understanding of the microbiota of the different segments of the gastrointestinal tract of chinchillas. The microbiota in the large intestine were significantly different to those in the proximal gastrointestinal segments. The cecum and colon exhibited the highest richness and diversity among all different segments of the gastrointestinal tract. The core microbiota are Firmicutes, Bacteroidota, Actinobacteriota, and Proteobacteria, with Firmicutes as the most abundant phylum in all the segments. Although there are similarities to the signature microbiota in other cecal fermenters, the Atopobiaceae family was first reported to be found in the gastrointestinal tract of cecal fermenters. Overall, this study provides a basis for further research on the effect of gastrointestinal microbiota upon the health of chinchillas. 

## Data Availability

The data presented in this study are openly available in Figshare at https://figshare.com/articles/dataset/Data/25035524 (accessed on 20 January 2024).
